# Diabetic retinopathy in Tanzania: prevalence and risk factors at entry into a regional screening programme

**DOI:** 10.1111/tmi.12652

**Published:** 2016-01-13

**Authors:** Charles R. Cleland, Matthew J. Burton, Claudette Hall, Anthony Hall, Paul Courtright, William U. Makupa, Heiko Philippin

**Affiliations:** ^1^ Eye DepartmentKilimanjaro Christina Medical CentreMoshiTanzania; ^2^ International Centre for Eye HealthLondon School of Hygiene & Tropical MedicineLondonUK; ^3^ Faculty of Psychology and NeuroscienceUniversity MaastrichtMaastrichtThe Netherlands; ^4^ Newcastle Eye Hospital Research FoundationWaratahNSWAustralia; ^5^ Kilimanjaro Centre for Community OphthalmologyUniversity of Cape TownCape TownSouth Africa

**Keywords:** diabetic retinopathy, screening, Africa, rétinopathie diabétique, dépistage, Afrique

## Abstract

**Objective:**

The number of adults with diabetes in sub‐Saharan Africa (SSA) is expected to almost double by 2035. This study investigated the prevalence of diabetic retinopathy (DR) and its risk factors at entry into a community‐based screening programme.

**Methods:**

All persons with diabetes screened for retinopathy at entry into a screening programme in Kilimanjaro Region, Tanzania between November 2010 and December 2014 were included. Fundus photographs were taken with a Topcon retinal camera following pupil dilation. Data were collected on BP, random blood sugar, duration of diabetes, BMI and visual acuity on entry.

**Results:**

A total of 3187 persons were screened for DR. The prevalence of any DR was 27.9% (95%CI 26.4–29.5%) with background diabetic retinopathy (BDR), pre‐proliferative diabetic retinopathy (PPDR) and proliferative diabetic retinopathy (PDR) having a prevalence of 19.1% (95% CI 17.7–20.4%), 6.0% (95%CI 5.2–6.8%) and 2.9% (95%CI 2.3–3.5%), respectively. Maculopathy was present in 16.1% (95%CI 14.8–17.4%) of participants. Multivariable logistic regression analysis for the presence of any DR found independent associations with duration of diabetes (*P* < 0.0001), systolic BP (*P* < 0.0001), random blood sugar (*P* < 0.0001) and attending a government hospital diabetic clinic (*P* = 0.0339).

**Conclusions:**

This study is the first to present data from a DR screening programme in SSA. The results will provide policymakers with data to aid planning of DR screening and treatment services in the African region. The study highlights the importance of managing comorbidities within DR screening programmes.

## Abstract (fr)

**Objectif:**

Le nombre d'adultes atteints de diabète en Afrique subsaharienne (ASS) devrait presque doubler d'ici 2035. Cette étude a investigué la prévalence de la rétinopathie diabétique (RD) et les facteurs de risque à l'entrée dans un programme de dépistage communautaire.

**Méthodes:**

Toutes les personnes atteintes de diabète, dépistées pour la rétinopathie à l'entrée dans un programme de dépistage dans la région du Kilimandjaro, en Tanzanie entre novembre 2010 et décembre 2014 ont été incluses. Les images du fond d’œil ont été obtenues avec une caméra rétinienne Topcon après dilatation de la pupille. Les données sur la pression artérielle, la glycémie aléatoire, la durée du diabète, l’IMC et l'acuité visuelle ont été recueillies à l'entrée.

**Résultats:**

Un total de 3187 patients ont été dépistés pour la RD. La prévalence de toute RD était de 27,9% (IC95%: 26,4 à 29,5%) avec la rétinopathie diabétique de base (RDB), la rétinopathie diabétique pré‐proliférative (RDPP) et la rétinopathie diabétique proliférative (RDP) ayant une prévalence de 19,1% (IC95%: 17,7 à 20,4%), 6,0% (IC95%: 5,2 à 6,8%) et 2,9% (IC95%: 2,3 à 3,5%), respectivement. La maculopathie était présente chez 16,1% (IC95%: 14,8‐17,4%) des participants. L'analyse de régression logistique multivariée pour la présence de toute RD a trouvé des associations indépendantes avec la durée du diabète (p <0,0001), la pression artérielle systolique (p <0,0001), la glycémie aléatoire (p <0,0001) et la visite à une clinique du diabète dans un hôpital gouvernemental (p = 0,0339).

**Conclusions:**

Cette étude est la première à présenter des données à partir d'un programme de dépistage de la RD en Afrique subsaharienne. Les résultats fourniront aux décideurs des données pour aider à la planification des services de dépistage et de traitement de la RD dans la région africaine. L’étude souligne l'importance de la prise en charge des co‐morbidités dans les programmes de dépistage de la RD.

## Abstract (es)

**Objetivo:**

Se espera que el número de adultos con diabetes en África subsahariana (ASS) prácticamente sea el doble en el 2035. Este estudio investiga la prevalencia de retinopatía diabética (RD) y sus factores de riesgo al entrar en un programa comunitario de cribado.

**Métodos:**

Se incluyeron todas las personas con diabetes a las que se les realizaron pruebas para retinopatía al entrar en un programa de cribado en la región de Kilimanjaro, Tanzania entre Noviembre 2010 y Diciembre 2014. Se tomaron fotografías del fondo del ojo con una cámara de retina Topcon después de dilatar la pupila. Se recogieron datos sobre presión sanguínea, glucemia aleatoria, duración de la diabetes, índice de masa corporal y agudeza visual al entrar.

**Resultados:**

Se realizó el cribado para RD a un total de 3187 personas. La prevalencia de cualquier RD era del 27.9% (IC 95% 26.4‐29.5%) con una prevalencia de la retinopatía diabética de base (RDB), retinopatía diabética no proliferativa (RDNP) y retinopatía diabética proliferativa (RDP) del 19.1% (IC 95% 17.7‐20.4%), 6.0% (IC 95% 5.2‐6.8%) y 2.9% (IC 95% 2.3‐3.5%), respectivamente. La maculopatía estaba presente en un 16·1% (IC 95% 14·8‐17·4%) de los participantes. Un análisis mediante regresión logística multivariable para la presencia de cualquier RD encontró asociaciones independientes con la duración de la diabetes (p<0.0001), presión sistólica (p<0.0001), glucemia aleatoria (p<0.0001) y el ser atendido en una clínica gubernamental para diabetes (p=0.0339).

**Conclusiones:**

Este estudio es el primero que presenta datos de un programa de cribado para RD en ASS. Los resultados proveerán a los políticos con datos que ayuden a planear los servicios de cribado y tratamiento de RD en la región africana. Este estudio subraya la importancia del manejo de comorbilidades dentro de los programas de cribado de RD.

## Introduction

Sub‐Saharan Africa (SSA) faces a rising epidemic of non‐communicable disease including heart disease, stroke, cancer and diabetes mellitus 1, 2, 3, 4. It is predicted that by 2030 non‐communicable diseases will cause 46% of all deaths in SSA driven by the changing demographic profile of the population and as a presumed consequence of increased urbanisation, with its associated lifestyle changes including diet, physical inactivity, smoking, alcohol use and obesity 5, 6, 7, 8.

The number of adults with diabetes mellitus (DM) in SSA is expected to almost double, from 21.5 million to 41.5 million, by 2035 9. Diabetic retinopathy (DR) is the commonest microvascular complication of DM and can lead to irreversible blindness 10. Vision loss can be prevented through tight glycaemic and BP control thus reducing microvasular damage and through the early detection and timely treatment of DR with laser photocoagulation and intravitreal agents 11. As DM and DR become more prevalent throughout the African region, it is important that strategies are developed to enable the early detection and adequate management of this emerging epidemic.

There are limited data available on the prevalence, incidence and progression of DR in SSA 12. There is strong evidence from Europe and the USA on the effect of glycaemic control and copathology such as hypertension on the development on DR. In contrast, there are few data from African populations that address these questions. To effectively plan and develop services to detect and treat DR in SSA, it is essential that the prevalence of DR and its associated risk factors within the African region be investigated.

The Kilimanjaro Diabetic Programme (KDP) was one of the first regional DR screening and treatment services to be established in SSA. We report estimates of the prevalence of DR and its associated risk factors among individuals with DM, at the time of their entry into this programme. As this is the first study to report data from a regional DR screening programme, the results are of relevance to those developing DR screening and treatment services elsewhere in SSA.

## Methods

The total population of Kilimanjaro Region of Northern Tanzania was reported as 1.64 million in the 2012 census 13. The population is distributed across an area of 13 250 km^2^. 24.2% of the population live in urban areas with an average household size of 4.3 persons. An estimated 9.7% of the population of Kilimanjaro Region is older than 60 years, the highest in Tanzania 13.

**Figure 1:**
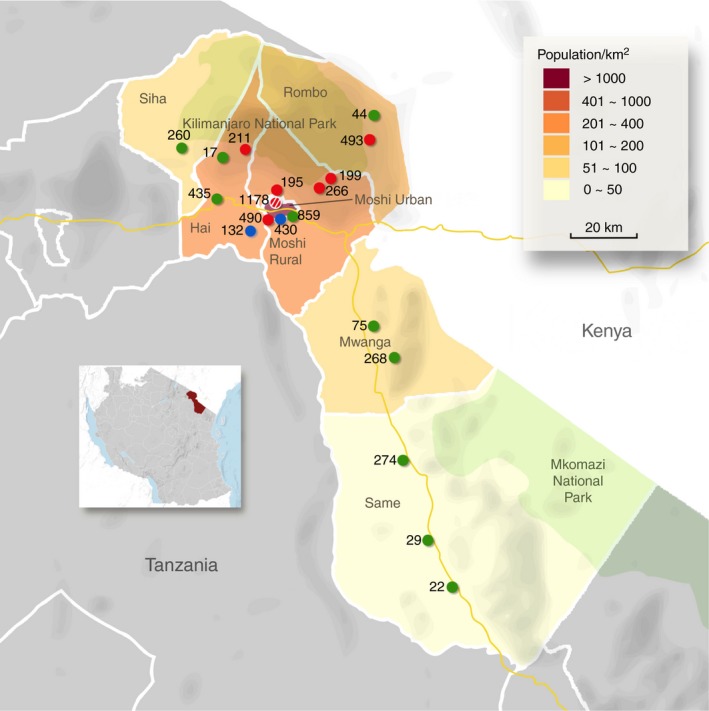
Map of Kilimanjaro Region and the diabetic clinics. The green dots indicate government hospitals, the red dots, church‐based hospitals, and the blue dots, private hospitals. The red and white striped dot indicates the referral hospital, Kilimanjaro Christian Medical Centre. The number next to each dot corresponds to the total number of persons with diabetes registered with the Kilimanjaro Diabetic Programme in each clinic.

There are 18 health facilities, including government, church‐based and private hospitals within Kilimanjaro Region providing services for people with diabetes (Figure 1). The Kilimanjaro Diabetic Programme (KDP) is an integrated clinic‐based mobile retinal screening service that has operated in Kilimanjaro Region since November 2010. All known persons with diabetes are registered with the KDP, after providing consent, following an appointment at one of the 18 diabetic clinics.

Following registration with the KDP, the patient's name, age, diabetic clinic, education level and residence are entered into a central database at Kilimanjaro Christian Medical Centre (KCMC) and they are assigned a unique KDP number. Patients were categorised as living in an urban or rural area based on residence information. There are seven districts in Kilimanjaro region and only patients residing in Moshi Urban District were classified as living in an urban area. Moshi is the largest town in Kilimanjaro region with a population of 184 292 13.

KCMC Eye Department is a tertiary referral centre for ophthalmology and provides a full range of services including laser photocoagulation and vitreoretinal surgery. Following registration with the KDP, patients are then informed when screening will take place and all persons registered with the KDP are invited to attend. The 18 diabetic clinics are each visited approximately one day per month on dedicated diabetic clinic days.

**Figure 2:**
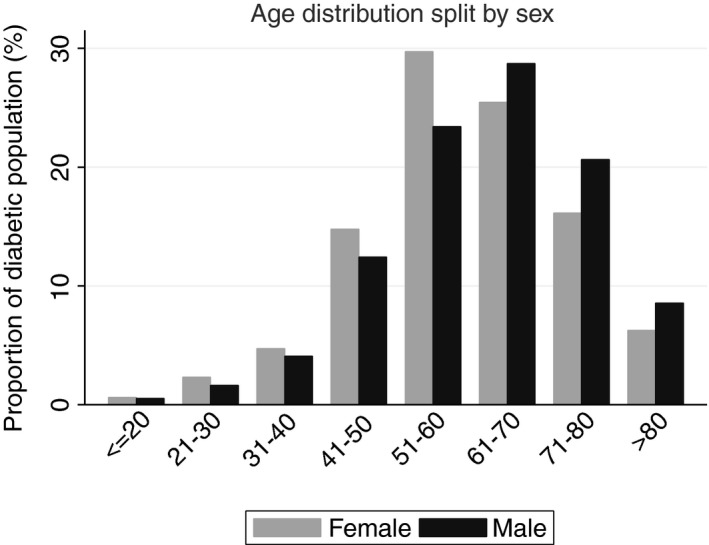
Age distribution of the KDP patients, subdivided by sex.

During DR screening visits, trained retinal photographers took fundus photographs of all patients using a Topcon retinal camera (TRC NW8S: Topcon Corporation, Tokyo, Japan). Two 45° images (disc‐centred and macula‐centred views) were taken in each eye; this could be with or without pupil dilation with topical g‐tropicamide 1% eye drops, depending on the quality of the view. Staff from the University of Birmingham (UK), through the Vision 2020 Links Programme, and an experienced ophthalmologist trained the retinal photographers. The images were stored on a laptop and later uploaded onto a backup hard‐drive. Ophthalmology residents (specialist eye doctors in training) at KCMC graded all fundus images during the retina component of their four‐year ophthalmology specialist training. All ophthalmology residents performing the grading were in either the third or fourth year of their ophthalmology specialist training. Two consultant vitroretinal surgeons (WM and AH) trained all ophthalmology residents on DR grading. Both received training on DR grading at the University of Birmingham (UK), through the Vision 2020 Links Programme. They randomly selected 10% of the images, which were regraded for quality control, and individual feedback was provided to graders on their performance. The minimum data set outlined in the English and Wales National Grading scheme was used for DR grading (Appendix Table A1) 14.

Images are classified in two ways: (i) degree of general retinopathy (no diabetic retinopathy, background diabetic retinopathy, pre‐proliferative retinopathy or proliferative retinopathy) and (ii) the degree of maculopathy (no maculopathy, non‐referable maculopathy or referable maculopathy) 14. Background DR is defined as any microaneurysms, retinal haemorrhages or exudates; pre‐proliferative retinopathy as any venous beading, venous loop, blot haemorrhages or intraretinal microvascular abnormalities; and proliferative retinopathy as any evidence of new vessel growth. Non‐referable maculopathy is defined as any microaneurysm or haemorrhage within 1 disc diameter of the centre of the fovea and referable maculopathy as any exudates within 1 disc diameter of the centre of the fovea (Appendix Table A1).

For this study, any DR was defined as the presence of any of the following: background DR, pre‐proliferative DR, proliferative DR, non‐referable maculopathy, referable maculopathy, previous panretinal photocoagulation, previous focal laser or any combination of the fore mentioned.

Patients found to have retinopathy requiring further investigations or treatment are referred to the KCMC Hospital Eye Department. They are phoned or sent a text message two weeks after their screening event by a member of the KDP team and informed that they need further investigations and possibly treatment and are advised when they should attend KCMC. Patients are advised to attend KCMC urgently or within 6 months. The thresholds for urgent referral are as follows: proliferative retinopathy, referable maculoapthy, an ungradable image or other pathology, for example a glaucomatous disc. Patients with pre‐proliferative retinopathy are advised to attend KCMC Eye Department within 6 months. Patients not requiring further investigation are informed of this via text message and advised to attend another screening event after 1 year.

Data on demographics, blood pressure, random blood sugar, duration of DM, height, weight, visual acuity and education level are collected during screening events. These data are recorded on a paper form and later entered into the central database at KCMC. All data in this paper were from the KDP database. No KCMC Hospital data were included.

### Statistical analysis

The electronic data were exported from the central KDP database (MS Access) and analysed using STATA version 13.0. The eye having the worse diabetic retinopathy was used for the analysis. Characteristics of the study population were described using means (SD) for continuous variables and absolute numbers with percentages for categorical variables. Differences in mean values were compared using the *t*‐test and proportions were compared with the chi‐squared test. Multivariable logistic regression analysis was used to investigate the relationship between diabetic retinopathy and a number of explanatory variables: age, gender, systolic BP, duration of DM, random blood sugar and clinic type. A backward stepwise selection procedure was used with a *P*‐value of 0.2 as the criterion for entry of the variables. A *P*‐value of <0.05 was considered to indicate statistical significance.

### Ethical approval

Ethical approval was obtained from the Kilimanjaro Christian Medical College University ethics committee.

## Results

### Diabetes mellitus in Kilimanjaro region

As of January 2015, a total of 5729 individuals were registered with the KDP. Of these, 3072 (53.6%), 1971 (34.4%) and 686 (12.0%) attended government, church and private hospitals, respectively. A total of 1221 (21.3%) were from the Moshi urban area, and 3452 (60.3%) were female. Registered females had a median age of 60 years (IQR 52–69) with a median duration of DM of 6 years (IQR 4–11). The median age of registered males was 63 years (IQR 54–72) with a mean duration of DM of 6 years (IQR 4–12) (Figure 2).

### Prevalence of diabetic retinopathy

By December 2014, of the 5729 individuals registered with the KDP, 3463 (60.4%) had been screened for DR. Screened patients represent all individuals registered with the KDP who attended a screening event and had fundus images taken. The demographic characteristics of individuals who had and had not been screened are shown in Table 1. Female gender, living in a rural area and attending a diabetic clinic in either a church or private hospital were all significantly associated with attendance for retinal screening. However, the Topcon camera was non‐functional for a cumulative total of approximately one year of the four that the KDP has been running.

**Table 1:** Characteristics of individuals who have been screened and those who have not been screened for diabetic retinopathy in Kilimanjaro Region, Tanzania

Characteristic	Not screened		Screened		OR	(95% CI)	*P*‐value
	*n*	(%)	*n*	(%)			
Total	2266	(39.6)	3463	(60.4)			
Female^a^	1264	(36.6)	2188	(63.4)	1.0	(reference)	
Male^a^	1002	(44.0)	1275	(56.0)	0.74	(0.66–0.82)	<0.0001
Mean age, years (SD)^b^	60.9	(15.2)	60.8	(13.0)	1.00	(0.99–1.00)	0.9087
Residence^a^							
Rural	1722	(38.2)	2786	(61.8)	1.0	(reference)	
Urban	544	(44.5)	677	(55.5)	0.77	(0.68–0.87)	0.0001
Education^c^							
No formal education	114	(40.4)	168	(59.6)	1.0	(reference)	
Primary‐level education	1139	(40.2)	1698	(59.8)	1.01	(0.79–1.30)	0.9278
Secondary‐level education	487	(41.6)	685	(58.4)	0.95	(0.73–1.24)	0.7300
College education	12	(48.0)	13	(52.0)	0.74	(0.32–1.67)	0.4619
Clinic type attended^a^							
Government	1461	(47.6)	1611	(52.4)	1.0	(reference)	
Missionary	638	(32.4)	1333	(67.6)	1.89	(1.68–2.13)	<0.0001
Private	167	(24.3)	519	(75.7)	2.82	(2.33–3.40)	<0.0001

a Data available for 5729.

b Data available for 5727.

c Data available for 4316.

Of the 3463 individuals screened, 276 images (8.0%) were ungradable due to cataract. Therefore, a total of 3187 people had their fundus images graded for DR (Table 2). The overall prevalence of any diabetic retinopathy within this group was 27.9% (95% CI 26.4%–29.5%), and the overall prevalence of maculopathy was 16.1% (95% CI 14.8%–17.4%). Proliferative DR was found in 2.9% (95% CI 2.3%–3.5%). The proportion of individuals with more advanced DR increased with a longer duration of DM and poorer blood glucose control, indicated by a random blood sugar of ≥11 (Table 3).

**Table 2:** Prevalence and grade of diabetic retinopathy and maculopathy, subdivided by gender. Individuals were classified by the eye with more advanced disease

Clinical stage	Total			Male			Female			*P*‐value
	*n*	(%)	(95% CI)	*n*	(%)	(95% CI)	*n*	(%)	(95% CI)	
Retinopathy										
None	2296	72.0	(70.5–73.6)	823	70.4	(67.8–73.0)	1473	73.0	(71.1–75.0)	
BDR	607	19.1	(17.7–20.4)	228	19.5	(17.2–21.8)	379	18.8	(17.1–20.5)	
PPDR	191	6.0	(5.2–6.8)	81	6.9	(5.5–8.4)	110	5.5	(4.5–6.4)	
PDR	93	2.9	(2.3–3.5)	37	3.1	(2.2–4.2)	56	2.8	(2.1–3.5)	0.2639
Total	3187			1169			2018			
Maculopathy										
None	2648	83.9	(82.6–85.2)	965	83.5	(81.3–85.6)	1683	84.1	(82.5–85.7)	
Non‐referable	143	4.5	(3.8–5.3)	61	5.3	(4.0–6.6)	82	4.1	(3.2–5.0)	
Referable	366	11.6	(10.5–12.7)	130	11.6	(9.4–13.1)	236	11.8	(10.4–13.2)	0.2904
Total	3157			1156			2001			

BDR, background diabetic retinopathy; PPDR, pre‐proliferative diabetic retinopathy; PDR, proliferative retinopathy. *P*‐values were calculated using by chi‐squared to test for differences between males and females in the stages of retinopathy and maculopathy.

**Table 3:** Prevalence and grade of diabetic retinopathy and maculopathy in relation to duration of diabetes and random blood glucose. Individuals were classified by the eye with more advanced disease

	Retinopathy								Maculopathy					
	Total	BDR *n*	(%)	PPDR *n*	(%)	PDR *n*	(%)	*P*‐value	Total	NRM *n*	(%)	RM *n*	(%)	*P*‐value
Duration of DM (years)														
0–5	1365	129	(9.5)	43	(3.2)	20	(1.5)		1351	32	(2.4)	64	(4.8)	
6–10	913	184	(20.2)	50	(5.5)	22	(2.4)		905	48	(5.3)	102	(11.3)	
11–15	466	127	(27.3)	40	(8.6)	26	(5.6)		463	28	(6.1)	88	(19.0)	
16–20	206	77	(37.4)	31	(15.1)	9	(4.4)		205	18	(8.8)	60	(29.3)	
>20	176	71	(40.3)	26	(14.8)	13	(7.4)	<0.0001	173	15	(8.7)	43	(24.9)	<0.0001
Total	3126								3097					
Random blood sugar														
<11	1658	280	(16.9)	97	(5.9)	29	(1.8)		1312	69	(4.2)	152	(9.3)	
≥11	1325	292	(22.0)	82	(6.2)	53	(4.0)	<0.0001	1644	67	(5.1)	185	(14.1)	<0.0001
Total	2983								2956					

DM, diabetes mellitus; BDR, background diabetic retinopathy; PPDR, pre‐proliferative diabetic retinopathy; PDR, proliferative retinopathy. NRM, non‐referable maculopathy; RM, referable maculopathy. *P*‐values were calculated by chi‐squared test to assess the effect of increasing duration of DM and random blood sugar on the stage of retinopathy and maculopathy.

Of the patients screened, 2985 (86.6%), 317 (9.2%) and 144 (4.2%) were categorised as having mild or no visual impairment, moderate visual impairment or blindness, respectively, as per WHO classification. Persons with any diabetic retinopathy were significantly more likely to have a visual acuity worse than 6 of 18 (Table 4). Education data were available on 2414 of those screened: 1733 (71.8%) had completed a primary‐level education or less. A total of 2535 (79.5%) of the patients screened were from rural areas. Patients from urban areas were significantly younger (*P* < 0.0001) and had a higher level of education (*P* < 0.0001).

**Table 4:** Characteristics of subjects with and without retinopathy and with and without maculopathy

Characteristic	No retinopathy		Any retinopathy		OR	(95% CI)	*P*‐value	No maculopathy		Any maculopathy		OR	(95% CI)	*P*‐value
Male, *n* (%)^a^	823	(70.4%)	346	(29.6%)	1.0	–	–	965	(83.5%)	191	(16.5%)	1.0	–	–
Female, *n* (%)^a^	1473	(73.0%)	545	(27.0%)	1.14	(0.97–1.33)	0.1164	1683	(84.1%)	318	(15.9%)	1.05	(0.86–1.27)	0.6426
Mean age, years (SD)^a^	59.7	(13.1%)	62.7	(11.8%)	1.02	(1.01–1.02)	<0.0001	60.0	(12.9%)	62.7	(11.5%)	1.02	(1.01–1.02)	<0.0001
Visual acuity <6/18, *n* (%)^b^	2112	(92.0%)	757	(85.0%)	2.04	(1.61–2.59)	<0.0001	2424	(91.6%)	422	(82.9%)	2.24	(1.71–2.93)	<0.0001
Mean DM duration, years (SD)^c^	7.0	(5.8%)	12.0	(7.6%)	1.11	(1.10–1.13)	<0.0001	7.6	(6.3%)	12.2	(7.5%)	1.09	(1.07–1.10)	<0.0001
Mean random blood glucose (SD) d	11.2	(5.5%)	12.3	(5.6%)	1.04	(1.02–1.04)	<0.0001	11.3	(5.6%)	12.6	(5.6%)	1.04	(1.02–1.06)	<0.0001
Mean systolic BP, mmHg (SD)^e^	132	(23.0%)	139	(24.7%)	1.01	(1.01–1.02)	<0.0001	133	(23.3%)	139	(24.9%)	1.01	(1.01–1.01)	<0.0001
Mean diastolic BP, mmHg (SD)^f^	83	(11.9%)	84	(12.6%)	1.0	(1.00–1.02)	0.0016	82.7	(11.9%)	84.6	(13.0%)	1.01	(1.00–1.02)	0.0014
Mean BMI (SD)^g^	26.6	(5.6%)	26.0	(4.9%)	0.98	(0.96–0.99)	0.0281	26.6	(5.6%)	25.5	(4.8%)	0.9	(0.93–0.99)	0.0022
Residence, *n* (%)														
Rural	1829	(72.2%)	706	(27.9%)	1.0	–	–	2103	(83.9%)	404	(16.1%)	1.0	–	–
Urban	467	(71.6%)	185	(28.4%)	1.03	(0.85–1.24)	0.7903	545	(83.9%)	105	(16.1%)	1.0	(0.79–1.27)	0.9808
Education, *n* (%)														
No formal education	99	(67.8%)	47	(32.2%)	1.0	–	–	119	(82.6%)	25	(17.4%)	1.0	–	–
Primary‐level education	1119	(70.5%)	468	(29.5%)	0.88	(0.61–1.27)		1294	(82.4%)	227	(17.6%)	0.84	(0.53–1.31)	
Secondary‐level education	473	(71.3%)	190	(28.7%)	0.85	(0.58–1.24)		558	(84.8%)	100	(15.2%)	0.85	(0.53–1.38)	
College education	11	(61.1%)	7	(38.9%)	1.34	(0.49–3.68)	0.6827	15	(83.3%)	3	(16.7%)	0.95	(0.26–3.54)	0.5779
Clinic type attended, *n* (%)														
Government	1059	(67.6%)	508	(32.4%)	1.0	–	–	1270	(81.8%)	282	(18.2%)	1.0	–	–
Missionary	904	(77.0%)	271	(23.0%)	0.62	(0.53–0.74)		1003	(86.2%)	160	(13.8%)	0.72	(0.58–0.89)	
Private	333	(74.8%)	112	(25.2%)	0.70	(0.55–0.89)	<0.0001	375	(85.0%)	66	(15.0%)	0.79	(0.59–1.06)	0.0058

*P*‐values are calculated from chi‐squared test for proportions and *t*‐test for means.

a Data available for 3187.

b Data available for 3186.

c Data available for 3126.

d Data available for 2983.

e Data available for 2999.

f Data available for 2997.

g Data available for 1863.

Of the 3463 patients screened, 2483 (71.7%) had been screened once for retinopathy over the four years that the KDP has been functioning. Of the remaining 980 patients, the number screened for retinopathy two, three, four and five times were 717 (20.7%), 227 (6.6%), 35 (1.0%) and 1 (0.03%), respectively. After grading of the baseline fundus images, 1297 (37.5%) patients were referred to KCMC for further investigation. Of those referred, 546 (42.1%) presented to KCMC Hospital. The patients who presented to KCMC following referral were more likely to be male (OR 1.34, 95% CI = 1.07–1.68) and were significantly more educated (OR 1.51, 95% CI = 1.21–1.88). There were no differences in age, category of referral hospital (government, church or private) or residing in an urban or rural area between those who complied with the follow‐up recommendation and those who did not.

### Associations with diabetic retinopathy

The demographic and clinical characteristics of individuals with and without any retinopathy are shown in Table 4. Those with any diabetic retinopathy were significantly older, had a higher blood pressure, a higher random blood sugar, a slightly lower BMI and were significantly more likely to attend a diabetic clinic in a government hospital. There were no significant differences in the presence of any stage of retinopathy or maculopathy or in DM control (indicated by random blood sugar) between patients from urban and rural locations.

Multivariable logistic regression analysis revealed that the duration of diabetes, random blood sugar, systolic blood pressure and attending a government hospital diabetic clinic were significantly associated with retinopathy (Table 5).

**Table 5:** Univariate and multivariable logistic regression analysis for factors associated with the presence of any diabetic retinopathy

Variable	Univariate analysis			Multivariable logistic regression		
	OR	95% CI	*P*‐value	OR	95% CI	*P*‐value
Duration of DM (years)						
0–5	1.0	–	–	1.0	–	–
6–10	2.38	1.93–2.94	<0.0001	2.29	1.83–2.86	<0.0001
11–15	4.32	3.40–5.49	<0.0001	3.94	3.05–5.09	<0.0001
16–20	8.03	5.86–11.01	<0.0001	7.57	5.40–10.63	<0.0001
>20	10.18	7.24–14.32	<0.0001	8.05	5.55–11.67	<0.0001
Systolic BP	1.01	1.01–1.02	<0.0001	1.01	1.01–1.01	<0.0001
Random blood sugar	1.04	1.02–1.05	<0.0001	1.04	1.03–1.06	<0.0001
Age	1.02	1.01–1.02	<0.0001	1.01	1.00–1.01	0.0746
Female sex	1.14	0.97–1.33	0.1164	1.06	0.88–1.28	0.5146
Body Mass Index	0.98	0.96–1.00	0.0283	Not included		
Clinic type						
Non‐government	1.0	–	–	1.0	–	–
Government	1.55	1.33–1.81	<0.0001	1.21	1.02–1.46	0.0339
Education				Not included		
No formal education	1.0					
Primary	0.88	0.61–1.27	0.4944			
Secondary	0.85	0.58–1.24	0.3960			
College	1.34	0.49–3.68	0.5694			
Residence				Not included		
Rural	1.0					
Urban	1.03	0.85–1.24	0.7903			

A number of individuals had missing data for the parameters entered in the multivariable logistic regression model. A large number of BMI measurements were missing from the data set, and therefore, BMI was excluded from the multivariable regression model. When BMI was included, attending a government hospital diabetic clinic was no longer significantly associated with the presence of DR. There were no other changes in inference.

## Discussion

This is the largest study to estimate the prevalence of diabetic retinopathy and its associated risk factors in diabetic patients in sub‐Saharan Africa and is the first to report data from a DR screening service in the region. In this population, the prevalence of any diabetic retinopathy was 27.9%, any maculopathy was 16.1%, proliferative retinopathy was 2.9%, pre‐proliferative retinopathy was 6.0%, and background diabetic retinopathy was 19.1%.

A recent systematic review of DR and maculopathy in Africa identified 62 studies of DR prevalence with only nine studies using retinal photographs to grade retinopathy (six of which were conducted in South Africa) 12. Three population‐based studies were identified in the African region, which estimated the prevalence range of DR as 30.2–31.6%, proliferative DR as 0.9–1.3% and any maculopathy as 1.2–4.5% 12. However, two of these studies were undertaken in Mauritius and Egypt (not in SSA) and the third is a population‐based study of visual impairment in adults >40 years old from Nigeria that did not grade DR or assess maculopathy. We identified one further population‐based survey from Kenya that was not included in the systematic review, which estimated the prevalence of any DR among persons with diabetes as 35.9%, macular oedema as 33.3% and vision‐threatening retinopathy (including clinically significant macular oedema, severe non‐proliferative DR and PDR) as 13.4% 15. The remaining studies in the systematic review were clinic‐based and largely from urban populations. The clinic‐based DR prevalence estimates were varied, with any DR ranging from 7.0 to 62.4%, proliferative DR from 0% to 6.9%, and any maculopathy from 1.2% to 31.1% 12.

The prevalence estimate of any maculoapthy from our data set is higher than the three population‐based estimates included in the systematic review but lower than the estimate from the survey in Kenya, at 16.1%. This could be explained by the fact that the definition of any maculopathy used in our programme and the definition used in the Kenyan survey included any haemorrhages within 1 disc diameter of the centre of the fovea, which could lead to false positives.

WHO estimates the prevalence of diabetes in adults aged 25–64 years in Tanzania as 9.1% 16. The number of persons in Tanzania aged 25–64 years is approximately 15.3 million 13, of whom an estimated 1.4 million have diabetes. If we extrapolate the results of this study that would suggest there are approximately 390 000 people aged 25–64 years with DR in Tanzania. Moreover, in this population, the prevalence of proliferative retinopathy and maculopathy, at 2.9% and 16.1%, respectively, are both higher than estimates from population‐based studies in SSA^12^ and estimates from Western populations 17, 18.

In a multivariable analysis, significant associations were found between the presence of DR and duration of DM, systolic BP, random blood sugar and attending a government diabetic clinic. The effects of copathology on DR development and progression have been more comprehensively studied in Western populations. Studies in the UK and USA have identified duration of diabetes 19, 20, high HbA1c levels 21, 22 and high blood pressure 23 as risk factors for DR. Duration of DM is an important factor to consider in the implementation of successful DR screening programmes across the region. In low‐income settings, an adjusted follow‐up regimen might be more feasible, with an emphasis on patients with a longer duration of DM. The recognition and treatment of suboptimal glycaemic control and hypertension are essential elements and are important educational messages for both patients and medical staff. Retinal screening activities need to be well integrated into the general care of persons with diabetes.

We found a significant correlation between attending a government hospital diabetic clinic and both lower attendance in the screening programme and an increased risk of any DR. A possible explanation for this association could be that patients attending government clinics are likely to be less affluent and less able to afford clinical services and medication. In SSA, the less well‐off rely mainly on the public sector for health care, are more likely to treat themselves and are less likely to use current preferred treatment options compared to the more well‐off 24. It is important that screening programmes in SSA develop strategies to ensure the inclusion of patients from all socioeconomic levels.

A further concern highlighted in this study was the low attendance (42.1%) at the central eye unit (KCMC) of patients who needed further assessment and treatment. Patients who were male and educated were more likely to follow the recommendation. As the burden of disease in SSA shifts to chronic diseases 25, it is essential that patients are regularly and reliably followed up. Further studies identifying the patient groups in which follow‐up is low, the reasons for poor follow‐up and the evaluation of strategies to improve follow‐up rates are needed.

The strengths of this study include the large number of people screened, the use of retinal photographs with a standardised retinopathy grading protocol and the mobile nature of the KDP covering a large geographical area, ensuring the inclusion of patients from both urban and rural populations across Kilimanjaro Region.

There are currently 5729 people registered with the KDP, however, only 3187 have been screened and graded for retinopathy. The main reason for this has been the necessity to move the retinal camera across the region on dirt roads, which has caused the camera to frequently break down. The camera is estimated to have been non‐functional for a cumulative total of one year. This will have inevitably been a contributory reason for a number of patients, although registered with the KDP, not being screened for DR. As these breakdowns occurred in a random manner and throughout the duration of the programme, we do not think this would have introduced any systematic selection or reporting bias. However, it does highlight the difficulties faced in accessing, maintaining and fixing expensive equipment in a low‐income setting. Alternative, low‐cost equipment deployed permanently to each remote clinic, which is easier to maintain and can be used by non‐specialists could increase the number of persons with diabetes who are screened for retinopathy. Although low‐cost alternative devices may increase coverage, it will be important to compare them to traditional fundus cameras in regards the proportion of ungradable images. In the KDP, when using a Topcon retinal camera, 8% of images were ungradable, which compares favourably to screening programmes in the UK 26.

Although two consultant vitreoretinal surgeons, who received additional training on grading DR in the UK, regraded 10% of the images for quality control, there are no data available on the outcomes of the quality control. As further DR screening services are established in SSA, it is important that adequate quality control is incorporated into screening programmes. This could be through partnership with accredited international reading centres. An additional limitation of this study is possible selection bias. In common with reports from all screening programmes, it is possible that the underlying prevalence of DR in persons with diabetes is lower. People who are known to the health system and who have been screened by the KDP will probably have had diabetes longer than those who have not.

The KDP measures random blood sugar during screening events and does not routinely collect data on HbA1c. Random blood sugar is a less sensitive marker for blood glucose control than HbA1c. However, random blood sugar has been suggested as an alternative to HbA1c in the African setting 27. The measurement of random blood sugar is cheaper, easier and more practical than HbA1C, especially during mobile screening events. Therefore, this could be considered as a practical option for estimating blood glucose control during screening programmes in a low‐income setting.

In conclusion, this is the largest study estimating the prevalence of DR and its associated risk factors in SSA and it is the first to publish data from a regional DR screening programme in SSA. The results emphasize the high disease burden Tanzania is likely to face from DR and provides important data for policymakers to aid in planning DR services in the wider region. Studies identifying the reasons for and strategies to improve follow‐up of patients after screening are necessary to effectively manage the growing burden of chronic disease in the African region.

## Appendix

**Table A1:** The Grading Scheme used in the Kilimanjaro Diabetic Programme based on the minimum data set recommended by the English and Wales National Screening Committee

Retinopathy (R)		
Level 0	None	
Level 1	Background	Microaneurysm(s)
		Retinal haemorrhage(s)
		Exudate(s)
Level 2	Pre‐proliferative	Venous beading
		Venous loop or reduplication
		Multiple deep, round or blot haemorrhages
		Intraretinal microvascular abnormality (IRMA)
Level 3	Proliferative	New vessels on the disc (NVD)
		New vessels elsewhere (NVE)
		Pre‐retinal or vitreous haemorrhage
		Pre‐retinal fibrosis
Maculopathy (M)		
No Maculopathy		Does not meet any of the criteria for maculopathy
Non‐referable maculopathy		Any microaneurysm or haemorrhage with 1 disc diameter (DD) of the fovea
Referable maculopathy		Any exudate within 1 DD of the centre of the fovea
Photocoagulation (P)		Focal/grid to macula
		Peripheral scatter
Ungradable (U)		Ungradable/unclassifiable
